# Chronic Olanzapine Treatment Induces Disorders of Plasma Fatty Acid Profile in Balb/c Mice: A Potential Mechanism for Olanzapine-Induced Insulin Resistance

**DOI:** 10.1371/journal.pone.0167930

**Published:** 2016-12-14

**Authors:** Huqun Li, Maosheng Fang, Mingzhen Xu, Shihong Li, Juan Du, Weiyong Li, Hui Chen

**Affiliations:** 1 Department of Pharmacy, Union Hospital Affiliated to Tongji Medical College, Huazhong University of Science and Technology, Wuhan, PR China; 2 Wuhan Mental Health Center, Wuhan, PR China; 3 College of Pharmacy, Hubei University of Chinese Medicine, Wuhan, PR China; 4 Department of Infectious Disease, Union Hospital Affiliated to Tongji Medical College, Huazhong University of Science and Technology, Wuhan, PR China; Max Delbruck Centrum fur Molekulare Medizin Berlin Buch, GERMANY

## Abstract

**Background:**

Atypical antipsychotics such as olanzapine cause metabolic side effects leading to obesity and insulin resistance. The underlying mechanisms remain elusive. In this study we investigated the effects of chronic treatment of olanzapine on the fatty acid composition of plasma in mice.

**Methods:**

Twenty 8-week female Balb/c mice were randomly assigned to two groups: the OLA group and the control group. After treatment with olanzapine (10 mg/kg/day) or vehicle intraperitoneally for 8 weeks, fasting glucose, insulin levels and oral glucose tolerance test were determined. Effects on plasma fatty acid profile and plasma indices of D5 desaturase, D6 desaturase and SCD1 activity were also investigated.

**Results:**

Chronic administration of olanzapine significantly elevated fasting glucose and insulin levels, impaired glucose tolerance, but did not increase body weight. Total saturated fatty acids and n-6 polyunsaturated fatty acids were significantly increased and total monounsaturated fatty acids were significantly decreased, while total n-3 polyunsaturated fatty acids showed no prominent changes. Chronic olanzapine treatment significantly up-regulated D6 desaturase activity while down-regulating D5 desaturase activity. Palmitic acid (C16:0), dihomo-γ-linolenic acid (C20:3n-6) and D6 desaturase were associated with an increase probability of insulin resistance, whereas nervonic acid (C24:1) and SCD1 were significantly associated with a lower insulin resistance probability.

**Conclusions:**

All results indicated that such drug-induced effects on fatty acid profile in plasma were relevant for the metabolic adverse effects associated with olanzapine and possibly other antipsychotics. Further studies are needed to investigate geneticand other mechanisms to explain how plasma fatty acids regulate glucose metabolism and affect the risk of insulin resistance.

## Introduction

Olanzapine is a widely used second generation antipsychotic (SGA) drug for the treatment of schizophrenia with a low propensity for neurological side effects[1]. However, it is frequently associated with serious metabolic side effects, such as dyslipidemia and insulin resistance (IR). Weight gain is frequently observed with olanzapine treatment in pre-clinical studies and in clinical settings[2–4]. However, the risk of metabolic side effects such as IR may be independent of weight gain[5]. Numerous studies have documented metabolic changes in the absence of substantial weight gain in clinical patients[6]. Furthermore, studies with healthy subjects showed that treatment with olanzapine caused significant metabolic impairments in the absence of weight gain[7–10]. Despite this body of evidence, the mechanisms underlying olanzapine-induced dyslipidemia and insulin resistance remain elucidated.

The fatty acids (FAs) in particular are highly associated with obesity, IR, and type 2 diabetes[11–15]. Several lines of evidence suggest that FAs are involved in the pathogenesis of IR via a reduction of insulin sensitivity and the promotion of pancreatic beta cell apoptosis and dysfunction[16–18]. Different FAs have distinct effects on insulin sensitivity, beta cell function andtissue inflammation[19]. In particular, n-6 polyunsaturated fatty acids (PUFAs) and saturated fatty acids (SFAs) (especially arachidonicacid and palmitic acid) can be viewed as pro-inflammatory molecules, whereas n-3 PUFAs (especially eicosapentaenoic acid and docosahexaenoic acid) can be viewed as anti-inflammatorymolecules[20].

The FA profile in blood partly reflects dietary FA intake, but it is also strongly determined by the endogenous FA metabolism[21]. The D5 desaturase (D5D) and D6 desaturase (D6D) catalyze the rate-limiting steps in the conversion of linoleic acid and α-linolenic acid into longchain n-6 and n-3PUFAs, whereas the stearoyl-CoAdesaturase-1 (SCD1) is required for the conversion of SFAs into monounsaturated fatty acids (MUFAs)[22]. By introducing a double bond into the FA chain, desaturaseenzymes have important consequences on thechemical structure of FAs and subsequently on their function. In fact, the activity of all three desaturaseshas been implicated in the development of certain metabolic diseases such as insulin resistance and type 2 diabetes[23–25].

The FA profile can be used as an indicator of disease risk[26]. An altered FA profile and estimated activities of desaturases have been associated with insulin resistance, type 2 diabetes[27], obesity[28], hypertriglyceridemia[29] and cardiovascular disease[30]. Free FAs concentrations are decreased following olanzapine treatment in clinical and animal studies[9]. However, previous basic and clinical studies also suggest that antipsychotics may augment PUFAs biosynthesis[31]. To our knowledge, there has not yet been an evaluation of the effect of chronic olanzapine treatment on the metabolic parameters and FA profile in Balb/c mice or human. Therefore, the primary aim of the current study was to establish, for the first time, the plasma FA profile in Balb/c mice after chronic treatment of olanzapine and to assess their associations with insulin resistance.

## Material and Method

### Animals

Twenty 8-week female Balb/c mice weighing 20–25g (Huafukang, Beijing, China) were purchased and maintained under a 12-hour light/dark cycle (lights on at 08:00 h) at 22±1°C. Animals were housed individually and allowed free access to food and water. Prior to the experiments, animals were routinely acclimated (>1 week) tolaboratory conditions to reduce potential stress effects during experiments. The animal facilities and protocol were performed in accordance with the guidelines of the Chinese Council on Animal Care and approved by the Institutional Animal Care and Use Committee of the Tongji Medical College, Huazhong University of Science and Technology (Permit Number:XH-B20150422).The number of mice was theminimum necessary to obtain significant results and inagreement with the triple R spirit for reduction of the number of animals used. Body weight was routinely recorded. Mice were anesthetized by isoflurane (Sigma, USA) and sacrificed at 9:00 am after 8 weeks of olanzapine treatment. Blood was collected into EDTA-coated tubes and plasma was isolated by centrifugation at 1,800×*g* for 10 min at 4°C. All samples were stored at –80°C until further analysis. All efforts were made to minimize suffering.

The primary study endpoints were the fasting insulin level and the homeostatic model assessment of insulin resistance (HOMA-IR) index. The secondary endpoint was the oral glucose tolerance test (OGTT).

### Drug

Mice were randomly assigned to two groups: the OLA group and the control group. The OLA group received olanzapine intraperitoneally (10mg/kg/day) in the morning (08:00–08:40h) for 8 weeks, while the control group received drug vehicle. All injection volumes were 10ml/kg.

Olanzapine (Sigma, USA) was dissolved in0.1 N HCl in distilled water, adjusting to pH 6.0 with 1 N NaOH, and adding distilled water to reach the desired concentration. Vehicle was similarly pH-adjusted, distilled water. The solutions were stored at 4°C and protected from light degradation. At the time of drug administration, body weight was measured.

### OGTT

OGTT was performed on the last day of the chronic treatment. Mice were food restricted for 14h prior to the OGTT. Mice received olanzapine one hour prior to the start of the OGTT. During the OGTT, basal blood samples and glucose measurements were obtained and then glucose was given via oral gavage (1.5g glucose/kg). Blood samples were collected via a tail snip at 30, 60, 90, and 120 min and glucose levels were determined. Blood glucose levels at 30, 60, 90, 120 min were determined via a tail snip method. Individual glucose measurements at the 5 time points during the OGTT were integrated to generate a single area under the curve (AUC) value.

### Plasma glucose and insulin

Fast glucose concentrations were determined using a hand-held glucometer (One Touch Ultra). Fast insulin levels were measured using commercially available kits (ELISA, Crystal Chem Inc., IL, USA) according to the manufacturer’s instructions. All analyses were performed by a technician blinded to treatment.

### Insulin resistance

To determine insulin resistance in mice, we calculated the HOMA-IR index on the last day of every week. This equation takes into account the product of both fasting levels of glucose (expressed as mmol/L) and insulin (μU/mL) at 60 minutes post-olanzapine treatment and divides by a constant of 22.5 ([I_0_ x G_0_]/22.5), where I_0_ and G_0_ are fasting insulin and glucose. A larger calculated HOMA-IR value denotes greater insulin resistance.

### Fatty acid analysis

The plasma FA profile was determined by gas chromatography with a previous derivatization to their corresponding fatty acid methyl esters[31]. Briefly, fatty acids were trans-esterified and analysed using a TSQ 8000 gas chromatography system (Thermo Fisher Scientific, USA). Fatty acid identification was based on retention times of authenticated fatty acid methyl ester standards (Sigma, USA). Results were expressed as weight percent of total fatty acids (mg fatty acid/100mg fatty acids). We calculated total SFAs, MUFAs, n-3 and n-6 PUFAs. Desaturase activity was estimated indirectly using FA product/precursor ratios[32]. D5 Desaturase activity was calculated as the ratio of C20:4n-6/C20:3n-6, D6 Desaturase activity was calculated as the ratio of C20:3n-6/C18:2n-6, and SCD1 activity was calculated asthe ratio of C18:1/C18:0.

### Statistics

All data are expressed as the mean±SD. To calculate statistical significance, Student’s *t*-test was used because our hypothesis is to test differences between two groups, the OLA group and the control group. The relationship between plasma FA composition, estimated desaturase activities and fasting glucose, insulin, HOMA-IR was determined by Spearman’s rank order correlation analysis. For allanalyses, statistical significance was determined at a *P*<0.05. Analyses were performed with SPSS version 15.0 (SPSS Inc.,Chicago, IL, USA).

## Results

**Fig 1:**
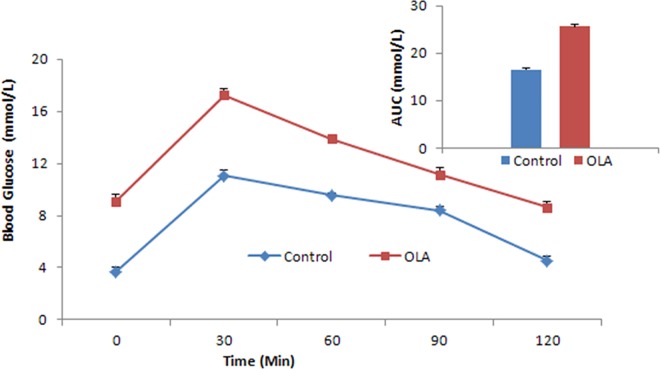
Effects of chronic olanzapine treatment on glucose and AUC(top right) levels in femaleBalb/c mice in the oral glucose tolerance test. Glucose levels of the OLA group were significantly elevated compared to the control group throughout the OGTT. The AUC of the OLA group was also significantly increased (*p*<0.001). AUC: area under the curve; OGTT: oral glucose tolerance test. * indicates different from the control group, *p*<0.001.

Mice in the OLA group exhibited a significant elevation in fasting glucose and insulin levels after 8-week treatment of olanzapine. In addition, a significant increase was evidenced in HOMA-IR index, suggesting an insulin resistance state in the OLA group. However, no significant change in body weight was observed after chronic olanzapine treatment compared to the control group (Table 1). After 8 weeks of olanzapine treatment, we challenged animals with an oral glucose tolerance test (Fig 1). The OLA group displayed significantly elevated fasting glucose and insulin levels after 14h of food restriction. Glucose levels were significantly increased compared to the control group throughout the OGTT. Additionally, the AUC was significantly increased by chronic olanzapine treatment (16.56mmol/L *vs* 25.59mmol/L, *p*<0.001), suggesting decreased whole-body insulin sensitivity. These results (detailed in S1 Data) demonstrate that chronically olanzapine administration induces insulin resistance without the change in body weight in mice.

**Table 1:** Mean value of weight, fasting glucose, insulin and HOMA-IR scores in the control group and the OLA group.

Variables	The control group			The OLA group		
	Baseline	8-week treatment	*p*	Baseline	8-week treatment	*p*
Weight(g)	21.27±0.38	21.43±0.35	0.3266	21.73±0.98	21.88±0.92	0.7257
G_0_	3.32±0.27	3.51±0.25	0.1151	3.49±0.39	8.37±0.48	<0.001
I_0_	23.35±0.43	23.25±0.68	0.6829	23.33±0.76	31.31±1.02	<0.001
HOMA-IR	3.44±0.26	3.62±0.24	0.1305	3.62±0.44	11.64±0.71	<0.001

G_0_, fasting glucose levels(mmol/L); I_0_, fasting insulin levels(μU/ml); HOMA-IR, homeostasis model assessment of insulin resistance(μU·mmol)/(ml·L).

Values are represented as mean±SD.

Plasma FAs composition are shown in Table 2.The plasma total SFAs (32.18% and 29.21%, *p*<0.001), and total n-6 PUFAs (29.34%and 26.10%, *p*<0.01) were both respectively higher in the OLA group compared to the control group. However, total MUFAs (34.60% and 40.88%, *p*<0.001) were significantly decreased in the OLA group compared to the control group. Total n-3 PUFAs showed no significant difference between two groups. In the OLA group, we observed increased palmitic acid (C16:0) and heptadecanoic acid (C17:0) levels when compared to the control group (20.29% and 18.74%, 0.70% and 0.52%). Linoleic acid (C18:2n-6), γ-linolenic acid (C18:3n-6), and dihomo-γ-linolenic acid (C20:3n-6) were statistically significantly increased in the plasma of OLA group as compared to the control group (22.1% and 19.46%, 0.55% and 0.47%, 1.26% and 0.90%), while eicosadienoic acid (C20:2n-6) was significantly decreased (0.77% and 1.01%). Moreover, in the OLA group myristoleic acid (C14:1), pentadecanoic acid (C15:1), palmitoleic acid (C16:1) and heptadecanoic acid (C17:1) were significantly decreased compared to the control group (0.09% and 0.14%, 0.02% and 0.03%, 4.72% and 6.85%, 0.39% and 0.44%), whereas nervonic acid (C24:1) was increased (0.07% and 0.05%). Our results (detailed in S1 Data) showed significantly decreased plasma activity of D5 desaturase and increased plasma activity of D6 desaturase in the OLA group compare to the control group. However, the plasma activity of SCD1 was similar in both groups.

**Table 2:** Fatty acid composition and estimated desaturase activities in mice after treatment.

Fatty acid	The control group		The OLA group		*p* value
	Mean	SD	Mean	SD	
C14:0, %	2.12	0.27	2.01	0.17	0.2797
C15:0, %	0.23	0.03	0.25	0.04	0.1776
C16:0, %	18.74	0.75	20.29	1.02	0.0011
C17:0, %	0.52	0.06	0.70	0.10	0.0001
C18:0, %	8.12	0.87	7.86	0.99	0.5289
C20:0, %	0.35	0.09	0.32	0.05	0.3286
C22:0, %	0.06	0.02	0.07	0.01	0.6426
C24:0, %	0.07	0.03	0.09	0.01	0.0751
Total SFAs, %	30.21	0.99	31.58	1.76	0.0462
C14:1, %	0.14	0.03	0.09	0.02	0.0002
C15:1, %	0.03	0.01	0.02	0.01	0.0023
C16:1, %	6.85	1.05	4.72	0.32	0.0000
C17:1, %	0.44	0.04	0.39	0.03	0.0123
C18:1, %	29.59	2.76	27.68	1.16	0.0591
C20:1, %	2.53	0.54	2.46	0.70	0.8144
C22:1, %	0.25	0.06	0.26	0.06	0.6124
C24:1, %	0.05	0.02	0.07	0.02	0.0114
Total MUFAs, %	39.88	1.85	35.70	0.95	0.0000
C18:3n-3, %	0.83	0.22	0.70	0.13	0.1074
C20:5n-3, %	0.51	0.16	0.53	0.08	0.6909
C22:5n-3, %	0.69	0.20	0.64	0.05	0.4356
C22:6n-3, %	1.76	0.31	2.03	0.28	0.0502
Total n-3 PUFAs, %	3.79	0.80	3.90	0.33	0.6986
C18:2n-6, %	19.46	1.21	22.01	2.55	0.0104
C18:3n-6, %	0.47	0.03	0.55	0.07	0.0031
C20:2n-6, %	1.01	0.09	0.77	0.12	0.0001
C20:3n-6, %	0.90	0.17	1.26	0.07	0.0000
C20:4n-6, %	3.82	0.46	3.56	0.39	0.1938
C22:4n-6, %	0.44	0.06	0.39	0.09	0.1568
Total n-6 PUFAs, %	26.10	1.42	28.54	2.20	0.0085
D5D(C20:4n-6/C20:3n-6)	4.32	0.69	2.83	0.21	0.0000
D6D(C20:3n-6/C18:2n-6)	0.05	0.01	0.06	0.01	0.0050
SCD1(C18:1/C18:0)	3.65	0.23	3.56	0.37	0.5255

D5D, D5 desaturase; D6D, D6 desaturase; SCD1, stearoyl coenzyme A desaturase-1.

Table 3 shows significant associations between FA proportions and estimated desaturase activities and fasting glucose, insulin levels and HOMA-IR in the OLA group (detailed in S1 Data). In the OLA group, plasma palmitic acid (C16:0) composition and dihomo-γ-linolenic acid (C20:3n-6) were positively correlated with insulin and IR, but not with glucose. Plasma heptadecanoic acid (C17:0), pentadecanoic acid (C15:1) and linoleic acid (C18:2n-6) were inversely correlated with insulin, but not with glucose or IR. Plasma nervonic acid (C24:1) was inversely with glucose and IR, but not with insulin. For estimated desaturase activities, D6 desaturase was positively associated with insulin and IR, whereas SCD1 was inversely associated with insulin and IR. D5 desaturase showed non-significant associations with glucose, insulin and IR.

**Table 3:** Spearman rank correlation coefficients between plasma fatty acids, estimated desaturase activities and fasting glucose, insulin and HOMA-IR in the OLA mice.

	Fasting glucose	Fasting insulin	HOMA-IR
Fatty acids			
C14:0	-0.49	0.45	-0.16
C15:0	-0.38	-0.72	-0.64
C16:0^#^	0.38	0.65*	0.68*
C17:0^#^	-0.28	-0.68*	-0.57
C18:0	0.31	0.79	0.70
C20:0	-0.50	-0.65	-0.80
C22:0	-0.63	-0.37	-0.79
C24:0	-0.54	0.68	-0.14
C14:1^#^	-0.28	0.21	-0.27
C15:1^#^	0.08	-0.72*	-0.28
C16:1^#^	0.26	0.26	0.18
C17:1^#^	-0.08	-0.38	-0.29
C18:1	0.49	0.54	0.65
C20:1	-0.29	-0.79	-0.68
C22:1	-0.66	-0.52	-0.89
C24:1^#^	-0.68*	-0.02	-0.66*
C18:3n-3	-0.45	-0.46	-0.61
C20:5n-3	0.54	0.47	0.69
C22:5n-3	-0.04	-0.48	-0.33
C22:6n-3	0.80	0.16	0.78
C18:2n-6^#^	-0.27	-0.83*	-0.61
C18:3n-6^#^	0.12	-0.22	-0.18
C20:2n-6^#^	-0.29	0.07	-0.40
C20:3n-6^#^	0.40	0.71*	0.68*
C20:4n-6	0.13	0.82	0.54
C22:4n-6	-0.18	0.53	0.02
D5D(C20:4n-6/C20:3n-6) ^#^	0.04	0.64	0.42
D6D(C20:3n-6/C18:2n-6) ^#^	0.42	0.76*	0.75*
SCD1(C18:1/C18:0)	-0.32	-0.82*	-0.73*

D5D, D5desaturase; D6D, D6desaturase; SCD1,stearoyl coenzyme A desaturase-1; HOMA-IR, homeostasis model assessment-insulin resistance.

^#^fatty acids and estimated desaturase activities that show significant difference between the OLA group and the control group.

*P<0.05.

## Discussion

The current study showed that chronic olanzapine treatment induces a significant increase of fasting glucose and insulin levels and insulin resistance in female Balb/c mice without weight gain. Mice in the OLA group showed higher levels of total SFAs and n-6 PUFAs and lower levels of total MUFAs than those in the control group. Particularly, the levels of FAs (C16:0), (C17:0), (C24:1), (C18:2n-6), (C18:3n-6) and (C20:3n-6) were higher, and the levels of FAs (C14:1), (C15:1), (C16:1), (C17:1) and (C20:2n-6) were lower in the OLA group compared to the control group. In contrast, the levels of total n-3 PUFAs showed no significant differences between the two groups. We also found that palmitic acid (C16:0), dihomo-γ-linolenic acid (C20:3n-6) and D6 desaturase were associated with an increase probability of IR, whereas nervonic acid (C24:1) and SCD1 were significantly associated with a lower IR probability.

Previous work has demonstrated that chronic treatment of SGAs frequently induces excessive weight gain and obesity in schizophrenic patients and animals[33]. However, in the present study we observed no significant weight gain in mice following chronic olanzapine treatment. In agreement with our results, Karen L.Teff et al[10] reported that olanzapine causes significant elevations in postprandial insulin, glucagon-like peptide 1, and glucagon coincident with insulin resistance in the absence of weight gain in healthy subjects. Moreover, decreased body weight has previously been observed in rats following chronic olanzapine or paliperidone treatment at specific dose[34]. Furthermore, numerous reports have documented hyperglycemia and new-onset type 2 diabetes in the absence of substantial weight gain inSGA-treated patients[6].

In the present study, we showed that chronic olanzapine treatment significantly increased total SFAs and decreased total MUFAs. Additionally, palmitic acid, the main contributor of total SFAs, was significantly increased and positively correlated with IR, in agreement with recent studies showing associations with impaired insulin sensitivity[35–36]. Many epidemiological reports suggested that diets high in saturated fats are associated with insulin resistance and an increased prevalence of type 2 diabetes[37]. In particular, elevated levels of SFAs induce inflammation, which results in insulin resistance via several pathways involving diacylglycerol-mediated protein kinase C activation or Toll-like receptors[38–39]. On the other hand, data are emerging which support that monounsaturated fatty acids have protective effects against saturated fat mediated toxicity[38–40]. Moreover, cultured cells incubated in high concentrations of saturated fatty acids exhibited impaired insulin signaling[41]. Interestingly, the addition of a monounsaturated fatty acid, such as oleate or palmitoleate, to palmitate in the incubation media can attenuate the deleterious effects of palmitate on insulin signaling[38]. The significant difference in the SFAs proportion between the OLA group and the control group that we observed was small(median 31.58% vs. 30.21%). However, the significant difference in the MUFAs proportion was obvious (median 35.70% vs. 39.88%). The biological relevance was unclear. It is possible that the deleterious effects of a high saturated fatty acid profile may be predominately mediatedvia impairments in other tissues (e.g. adipose, liver, vascular) and/or secondary to resulting pro-inflammatory/stress responses in these other tissues. Moreover, our results suggest that the higher proportion of SFAs and lower proportion of MUFAs in the OLA group may be caused by endogenous transformation of SFAs to MUFAs, as the calculated activity of SCD-1 was slightly lower in the OLA group.

At an exploratory level of significance we also found higher levels of the essential fatty acid linoleic acid (C18:2n-6) and total n-6 PUFAs in the OLA group. These n-6 PUFAs are linked to inflammatory signaling, insulin resistance and type 2 diabetes risk[42]. Therefore a linoleic acid (C18:2n-6) poor diet might have the beneficial effect of less arachidonic acid (C20:4n-6) production and anti-inflammatory eicosanoid synthesis[43]. In the current study, we found no significant difference in n-3 fatty acids between the OLZ groups and the control group. Consistent with our findings, Robert K. McNamara et al. showed that chronic treatment with olanzapine and quetiapine did not significantly up-regulate plasma indices of n-3 PUFAs biosynthesis[44]. Also in agreement with previous reports, n-3 PUFAs were not associated with the worsening of hyperglycemia or the risk of insulin resistance[36].

The FA profile in blood and tissues partly reflectsdietary FA intake, but it is also strongly determined bythe endogenous FA metabolism. The fatty acid-modifying enzymes for which connections to insulin resistance and type 2 diabetes have been shown include D5 desaturase, D6 desaturase and SCD[22]. Clinical studies have found that chronic treatment with risperidone or olanzapine significantly increase plasma indices of D6 desaturase activity[45]. Similarly, our results showed that chronic treatment of olanzapine significantly stimulated the activity of D6 desaturase. Moreover, the activity of D6 desaturase was found to positively correlate with insulin resistance, which was in agreement with previous studies[26, 46]. Our results also exhibited that chronic olanzapine treatment significantly decreased the activity of D5 desaturase. However, we did not observe significant difference in the activity of SCD1 between the two groups. Although correlations between the activity of D5 desaturase and SCD1 and insulin resistance were shown in previous studies[22, 26], we were unable to reproduce these observations, probably due to the different study animals.

Recently, it has been postulated that potential alterations in the metabolic pathway of PUFAs synthesis could constitute a fundamental trigger in the initiation and propagation of metabolic abnormalities, such as IR[26, 47]. Numerous previous studies reported that SGAs medications up-regulate long-chain PUFA biosynthesis in rats[44], and PUFAs reduce SCD1 mRNA expression at the level of transcription and mRNA stability[48–50]. SCD1 is of particular interest because it is the rate-limiting step in the transformation of pro-inflammatory SFAs to MUFAs[22]. In the present study, we also found that chronic treatment of olanzapine significantly increased n-6 PUFAs. Additionally, total SFAs was increased and total MUFAs was decreased in the OLA group. As SCD1 is the rate-limiting step in the transformation of SFAs to MUFAs, it is important to account for PUFAs in future antipsychotic studies. It is therefore possible that, at least in part, the side effects of chronic olanzapine treatment maybe related to changes in the n-6 PUFAs. Future studies with larger samples and different antipsychotic medications are needed to address this important question.

This study has three notable limitations. First, olanzapine has a shorter plasma half-life in rats (~2.5h) than in humans. However, previous studies showed that 15 days of i.p. olanzapine at 6 mg/kg body weight in rats produced a mean plasma concentration of 12.0±4.9 ng/ml at 3h after the last daily dose, which is comparable to the plasma concentration (above 9 ng/ml) that had a greater likelihood of clinical response[51]. Moreover, numerous reports showed that chronic daily treatment with olanzapine induced significant changes in adiposity and lipid metabolismin female rats[34,44,52]. Nevertheless, without the plasma olanzapine concentration data it remains possible that greater changes in FA profile may have been observed with a different mode of administration. Second, this study examined the insulin resistance in mice with OGTT test rather than clamp technique, which is the “gold-standard” technique. However, Heidi N. Boyda et al showed a high degree of correlation between results obtained with the GTT and the HIEC in rats treated with SGA drugs[53]. The OGTT test in this study may predict, at least in part, the insulin resistance state in mice. Third, due to the limitation of the lab, we put animals in adjacent rooms. However, throughout the experiment mice were treated in the same way, except in adjacent rooms. Nevertheless, in view of experiment design it would be better if the mice were put in the same room.

In conclusion, our study shows that chronic olanzapine treatment induced a significant increase in fasting glucose and insulin levels and insulin resistance without body weight gain. Chronic treatment of olanzapine significantly increased total SFAs and total n-6 PUFAs, while decreased total MUFAs. Olanzapine also significantly up-regulated D6 desaturase activity while down-regulated D5 desaturase activity. Our study also find that palmitic acid (C16:0), dihomo-γ-linolenic acid (C20:3n-6) and D6 desaturase were associated with an increase probability of IR, whereas nervonic acid (C24:1) and SCD1 were significantly associated with a lower IR probability. The present data provide further support for olanzapine-mediated perturbation of fatty acid profile in plasma as a molecular mechanism involved in antipsychotic-associated metabolic adverse effects. Further studies are needed to investigate genetic and other mechanisms to explain how plasma fatty acids regulate glucose metabolism and affect the risk of IR.

## Supporting Information

S1 Data Raw data between the control group and the OLA group for analysis and manuscript. (XLSX)

## References

[1] Lieberman JA, Stroup TS, McEvoy JP, Swartz MS, Rosenheck RA, Perkins DO, et al Effectiveness of antipsychotic drugs in patients with chronic schizophrenia. N Engl J Med. 2005;353(12):1209–1223. 10.1056/NEJMoa051688 16172203

[2] Shobo M, Yamada H, Mihara T, Kondo Y, Irie M, Harada K, et al Two models for weight gain and hyperphagia as side effects of atypical antipsychotics in male rats: validation with olanzapine and ziprasidone. Behav Brain Res. 2011;216(2):561–568. 10.1016/j.bbr.2010.08.046 20816897

[3] Cooper GD, Pickavance LC, Widling JP, Halford JC, Goudle AJ. A parametric analysis of olanzapine-induced weight gain in female rats. Psychopharmacology (Berl). 2005;181(1):80–89. 15778884 10.1007/s00213-005-2224-4

[4] Baptista T, ElFakih Y, Uzcategui E, Sandia I, Talamo E, Araujo de Baptista E, et al Pharmacological management of atypical antipsychotic-induced weight gain. CNS Drugs. 2008;22(6):477–495. 18484791 10.2165/00023210-200822060-00003

[5] Hahn M, Chintoh A, Giacca A, Xu L, Lam L, Mann S, et al Atypical antipsychotics and effects of muscarinic, serotonergic, dopaminergic and histaminergic receptor binding on insulin secretion in vivo: an animal model. Schizophr Res. 2011;131(1–3):90–95. 10.1016/j.schres.2011.06.004 21696923

[6] Newcomer JW. Second-generation (atypical) antipsychotics and metabolic effects: a comprehensive literature review. CNS Drugs. 2005;19 Suppl 1:1–93. 10.2165/00023210-200519001-00001 15998156

[7] Sacher J, Mossaheb N, Spindelegger C, Klein N, Geiss-Granadia T, Sauermann R, et al Effects of olanzapine and ziprasidone on glucose tolerance in healthy volunteers. Neuropsychopharmacology.2008;33(7):1633–1641. 10.1038/sj.npp.1301541 17712347

[8] Vidarsdottir S, de Leeuw van Weenen JE, Frolich M, Roelfsema F, Romijn JA, Pijl H. Effects of olanzapine and haloperidol on the metabolic status of healthy man. J Clin Endocrinol Metab. 2010;95(1):118–125. 10.1210/jc.2008-1815 19906788

[9] Albaugh VL, Singareddy R, Mauger D, Lynch CJ. A double blind,placebo-controlled, randomized crossover study of the acute metabolic effects of olanzapine in healthy volunteers. PLoS One. 2011;6(8):e22662 10.1371/journal.pone.0022662 21857944 PMC3153475

[10] Teff KL, Rickels MR, Grudziak J, Fuller C, Nguyen HL, Rickels K. Antipsychotic-Induced Insulin Resistance and Postprandial Hormonal Dysregulation Independent of Weight Gain or Psychiatric Disease. Diabetes. 2013;62:3232–3240. 10.2337/db13-0430 23835329 PMC3749337

[11] Tan CY, Virtue S, Murfitt S, Roberts LD, Phua YH, Dale M, et al Adiposetissue fatty acid chain length and mono-unsaturation increases with obesity and insulin resistance. Sci Rep. 2016;6:23873 10.1038/srep23873 27027977 PMC4813347

[12] Simopoulos AP. An increase in the omega-6/omega-3 fatty acid ratio increases the risk for obesity. Nutrient. 2016;8(3):128. 10.3390/nu8030128 PMC4808858 26950145

[13] Chen X, Xu S, Wei S, Deng Y, Li Y, Yang F, et al Comparative Proteomic Study of Fatty Acid-treated Myoblasts Reveals Role of Cox-2 in Palmitate-induced Insulin Resistance. Sci Rep. 2016;6:21454 10.1038/srep21454 26899878 PMC4761885

[14] Han E, Yun Y, Kim G, Lee YH, Wang HJ, Lee BW, et al Effects of Omega-3 Fatty Acid Supplementation on Diabetic Nephropathy Progression in Patients with Diabetes and Hypertriglyceridemia. PLoS One. 2016;11(5):e0154683 10.1371/journal.pone.0154683 27135947 PMC4852914

[15] Salto LM, Bu L, W. Beeson WL, Firek A, Cordero-MacIntyre Z, De Leon M. The Ala54Thr Polymorphism of the Fatty Acid Binding Protein 2 Gene Modulates HDL Cholesterol in Mexican-Americans with Type 2 Diabetes. Int J Environ Res Public Health. 2016;13(1):52. 10.3390/ijerph13010052 PMC4730443 26703680

[16] McGarry JD. Banting lecture 2001: dysregulation of fatty acid metabolism in the etiology of type 2 diabetes. Diatbete. 2002;51(1):7–18. 10.2337/diabetes.51.1.7 11756317

[17] Kashyap S, Belfort R, Gastaldelli A, Pratipanawatr T, Berria R, Pratipanawatr W, et al A Sustained Increase in Plasma Free Fatty Acids Impairs Insulin Secretion in Nondiabetic Subjects Genetically Predisposed to Develop Type 2 Diabetes. Diabetes. 2003;52(10):2461–2474. 14514628 10.2337/diabetes.52.10.2461

[18] Roden M. Does endurance training protect from lipotoxicity? Diabetes. 2012; 61(10):2397–2399. 10.2337/db12-0662 22997429 PMC3447918

[19] Hara T, Kimura I, Inoue D, Ichimura A, Hirasawa A. Free fatty acid receptors and their role in regulation of energy metabolism. Reviews of physiology, biochemistry and pharmacology. 2013;164:77–116. 10.1007/112_2013_13 23625068

[20] Sears B, Perry M. The role of fatty acids in insulin resistance.Lipids in Health and Disease. 2015;14:121 10.1186/s12944-015-0123-1 26415887 PMC4587882

[21] Bjermo H, Riserus U. Role of hepatic desaturases in obesity-related metabolic disorders. CurrOpinClinNutrMetab Care. 2010;13:703–708. 10.1097/MCO.0b013e32833ec41b 20823776

[22] Kroger J, Schulze MB. Recent insights into the relation of D5 desaturase and D6 desaturase activity to the development of type 2 diabetes.CurrOpinLipidol. 2012;23:4–10. 10.1097/MOL.0b013e32834d2dc5 22123669

[23] Krachler B, Norberg M, Eriksson JW, Hallmans G, Johansson I, Vessby B, et al Fatty acid profile of the erythrocyte membrane preceding development of type 2 diabetes mellitus. NutrMetabCardiovasc Dis. 2008;18:503–510. 10.1016/j.numecd.2007.04.005 18042359

[24] Chow LS, Li S, Eberly LE, Seaquist ER, Eckfeldt JH, Hoogeveen RC, et al Estimated plasma stearoyl co-A desaturase-1 activity and risk of incident diabetes: the Atherosclerosis Risk in Communities (ARIC) study. Metabolism. 2013;62:100–108. 10.1016/j.metabol.2012.06.004 22819528 PMC3518662

[25] Jacobs S, Schiller K, Jansen EH, Boeing H, Schulze MB, Kroger J. Evaluation of various biomarkers as potential mediators of the association between D5 desaturase, D6 desaturase, and stearoyl-CoA desaturase activity and incident type 2 diabetes in the European Prospective Investigation into Cancer and Nutrition–Potsdam Study. Am J ClinNutr. 2015;102:155–164. 10.3945/ajcn.114.102707 25971719

[26] Mayneris-Perxachs J, Guerendiain M, Castellote Al, Estruch R, Covas MI, Fito M, et al Plasma fatty acid composition, estimated desaturase activities, and their relation with the metabolic syndrome in a population at high risk of cardiovascular disease. Clinical Nutrition. 2014;33:90–97. 10.1016/j.clnu.2013.03.001 23591154

[27] Risérus U, Willett WC, Hu FB. Dietary fats and prevention of type 2 diabetes.Prog Lipid Res. 2009;48:44–51. 10.1016/j.plipres.2008.10.002 19032965 PMC2654180

[28] Warensjö E, Öhrvall M, Vessby B. Fatty acid composition and estimated desaturase activities are associated with obesity and lifestyle variables in men and women. NutrMetabCardiovasc Dis. 2006;16:128–136. 10.1016/j.numecd.2005.06.001 16487913

[29] Paillard F, Catheline D, Duff FL, Bouriel M, Deugnier Y, Pouchard M, et al Plasma palmitoleic acid, a product of stearoyl-coAdesaturase activity, is an independent marker of triglyceridemia and abdominal adiposity. NutrMetabCardiovasc Dis. 2008;18:436–440. 10.1016/j.numecd.2007.02.017 18068341

[30] Erkkilä A, de Mello VD, Risérus U, Laaksonen DE. Dietary fatty acids and cardiovascular disease: an epidemiological approach. Prog Lipid Res. 2008;47:172–187. 10.1016/j.plipres.2008.01.004 18328267

[31] McNamara RK, Able JA, Jandacek R, Rider T, Tso P. Chronic Risperidone Treatment Preferentially Increases Rat Erythrocyte and Prefrontal Cortex Omega-3 Fatty Acid Composition: Evidence for Augmented Biosynthesis. Schizophr Res. 2009;107(2–3):150–157. 10.1016/j.schres.2008.09.027 18993032 PMC2662584

[32] Mayneris-Perxachs J, Guerendiain M, Castellote AI, Estruch R, Covas MI, Fito M, et al Plasma fatty acid composition, estimated desaturase activities, andtheir relation with the metabolic syndrome in a population at highrisk of cardiovascular disease. ClinNutr. 2014;33(1):90–97. 10.1016/j.clnu.2013.03.001 23591154

[33] Henderson DC. Weight gain with atypical antipsychotics: evidence and insights. J Clin Psychiatry.2007; 68:18–26. 17956152

[34] McNamara RK, Jandacek R, Rider T, Tso P, Cole-Strauss A, Lipton JW. Atypical Antipsychotic Medications Increase Postprandial Triglyceride and Glucose Levels in Male Rats: Relationship with Stearoyl-CoA Desaturase Activity. Schizophr Res. 2011;129(1):66–73. 10.1016/j.schres.2011.03.016 21474290 PMC3100393

[35] Ma W, Wu JH, Wang Q, Lemaitre RN, Mukamal KJ, Djousse L, et al Prospective association of fatty acids in the de novo lipogenesis pathway with risk of type 2 diabetes: the Cardiovascular Health Study. Am J ClinNutr. 2015;101:153–163. 10.3945/ajcn.114.092601 PMC4266885 25527759

[36] Lankinen MA, Stančáková A, Uusitupa M, Ågren J, Pihlajamäki J, Kuusisto J, et al Plasma fatty acids as predictors of glycaemia and type 2 diabetes. Diabetologia. 2015;58(11):2533–2544. 10.1007/s00125-015-3730-5 26277381

[37] Hu FB, van Dam RM, Liu S. Diet and risk of Type II diabetes: the role of types of fat and carbohydrate. Diabetologia. 2001; 44(7):805–817. 10.1007/s001250100547 11508264

[38] Coll T, Eyre E, Rodríguez-Calvo R, Palomer X, Sánchez RM, Merlos M, et al Oleate reverses palmitate-induced insulin resistance and inflammation in skeletal muscle cells. J Biol Chem. 2008;283(17):11107–11116. 10.1074/jbc.M708700200 18281277

[39] Senn JJ. Toll-like receptor-2 is essential for the development of palmitate -induced insulin resistance in myotubes. J Biol Chem. 2006;281(37):26865–26875. 10.1074/jbc.M513304200 16798732

[40] Pardo V, González-Rodríguez A, Guijas C, Balsinde J, Valverde AM. Opposite Cross-Talk by Oleate and Palmitate on Insulin Signaling in Hepatocytes through Macrophage Activation.J Biol Chem. 2015;290(18):11663–11677. 10.1074/jbc.M115.649483 25792746 PMC4416868

[41] Chavez JA, Summers SA. Characterizing the effects of saturated fatty acids on insulin signaling and ceramide and diacylglycerol accumulation in 3T3-L1 adipocytes and C2C12 myotubes. Arch BiochemBiophys. 2003;419(2):101–109. 10.1016/j.abb.2003.08.020 14592453

[42] Calder PC. Fatty acids and inflammation: the cutting edge between food and pharma. European journal of pharmacology. 2011;668 Suppl 1:S50–58. 21816146 10.1016/j.ejphar.2011.05.085

[43] Patterson E, Wall R, Fitzgerald GF, Ross RP, Stanton C. Health implications of high dietary omega-6 polyunsaturated Fatty acids. J Nutr and Metab. 2012; 2012:539426. 22570770 10.1155/2012/539426 PMC3335257

[44] McNamara RK, Jandacek R, Rider T, Tso P, Cole-Strauss A, Lipton JW. Differential Effects of Antipsychotic Medications on Polyunsaturated Fatty Acid Biosynthesis in Rats: Relationship with Liver Delta6-Desaturase Expression. Schizophr Res. 2011;129(1):57–65. 10.1016/j.schres.2011.03.006 21458237 PMC3100388

[45] Kaddurah-Daouk R, McEvoy J, Baillie RA, Lee D, Yao JK, Doraiswamy PM, et al Metabolomic mapping of atypical antipsychotic effects in schizophrenia. Mol Psychiatry. 2007;12(10):934–945. 10.1038/sj.mp.4002000 17440431

[46] Sethom MM, Fares S, Feki M, Hadj-Taieb S, Elasmi M, Omar S, et al Plasma fatty acids profile and estimated elongase and desaturases activities in Tunisian patients with the metabolic syndrome. Prostaglandins LeukotEssent Fatty Acids. 2011;85(3):137–141. 10.1016/j.plefa.2011.06.006 21782403

[47] Araya J, Rodrigo R, Pettinelli P, Arava AV, Poniachik J, Videla LA. Decreased liver fatty acid delta-6 and delta-5 desaturase activity in obese patients.Obesity (Silver Spring). 2010;18(7):1460–1463. 19875987 10.1038/oby.2009.379

[48] Ntambi JM. Regulation of stearoyl-CoA desaturase by polyunsaturated fatty acids and cholesterol. J Lipid Res. 1999;40:1549–1558. 10484602

[49] Sessler AM, Kaur N, Palta JP, Ntambi JM. Regulation of stearoyl-CoA desaturase 1 mRNA stability by polyunsaturated fatty acids in 3T3-L1 adipocytes. J Biol Chem. 1996;271:29854–29858. 8939925 10.1074/jbc.271.47.29854

[50] Nakamura MT, Nara TY. Structure, function, and dietary regulation of delta6, delta5, and delta9 desaturases. Annu Rev Nutr. 2004;24:345–376. 10.1146/annurev.nutr.24.121803.063211 15189125

[51] Aravagiri M, Teper Y, Marder SR. Pharmacokinetics and tissue distribution of olanzapine in rats. Biopharm Drug Dispos. 1999;20(8):369–377. 10870093 10.1002/1099-081x(199911)20:8<369::aid-bdd200>3.0.co;2-6

[52] Mann S, Chintoh A, Giacca A, Fletcher P, Nobrega J, Hahn M, et al Chronic olanzapine administration in rats: effect of route of administration on weight, food intake and body composition.PharmacolBiochemBehav. 2013;103(4):717–722. 10.1016/j.pbb.2012.12.002 23234835

[53] Boyda HN, Procyshyn RM, Pang CC, Hawkes E, Wong D, Jin CH, et al Metabolic side-effects of the novel second-generation antipsychotic drugs asenapine and iloperidone: a comparison with olanzapine.PLoS One. 2013;8(1):e53459 10.1371/journal.pone.0053459 23326434 PMC3541274

